# Divergent aortic root cusp-to-cusp measurement techniques on CMR: geometric implications and threshold reclassification

**DOI:** 10.1093/ehjimp/qyag173

**Published:** 2026-10-12

**Authors:** Jo M Zelis, Arie van Dijk, Toon D Duijnhouwer, Khibar Salah, Robin H Heijmen, Robin Nijveldt

**Affiliations:** Cardiology Department, Radboud University Medical Center, Nijmegen, The Netherlands; Cardiology Department, Radboud University Medical Center, Nijmegen, The Netherlands; Cardiology Department, Radboud University Medical Center, Nijmegen, The Netherlands; Radiology Department, Radboud University Medical Center, Nijmegen, The Netherlands; Cardiothoracic Surgery Department, Radboud University Medical Center, Nijmegen, The Netherlands; Cardiology Department, Radboud University Medical Center, Nijmegen, The Netherlands

## Abstract

**Objective:**

To evaluate discrepancies between current European guidelines (ESC vs. EACTS) regarding aortic root cusp-to-cusp measurements and assess their impact on risk stratification for prophylactic root replacement.

**Methods and results:**

A retrospective analysis was performed on a relative young cohort of 50 patients (mean age 42 years) who underwent non-contrast 3D cardiovascular magnetic resonance (CMR) imaging. We compared the widest-to-widest cusp measurements and the middle cusp-to-cusp measurements with each other and against a traced aortic root perimeter. The widest cusp-to-cusp measurement yielded systematically higher values compared to the middle measurement, showing a significant mean difference of 3.60 mm (*P* < 0.001). The middle measurement demonstrated better statistical agreement with the traced aortic root perimeter, achieving a Lin’s Concordance Correlation Coefficient (CCC) of 0.98 and a Mean Absolute Error (MAE) of 3.96 mm. Conversely, widest cusp-to-cusp measurements exhibited substantially lower agreement (CCC 0.80) and significantly higher error rates (MAE 16.88 mm), consistently overestimating the perimeter. This led to the upward reclassification of 10.0% (5/50) and 16.0% (8/50) of patients across the 40 and 45 mm guideline thresholds, respectively.

**Conclusion:**

Cusp-to-cusp measurement techniques recommended by ESC and EACTS produce systematically divergent aortic root dimensions, resulting in significant patient reclassification across clinical thresholds. Although the middle measurement aligns more closely with the traced anatomical perimeter, the principal finding of this study is that guideline choice fundamentally alters patient categorization. Standardization of aortic root measurement conventions is essential to ensure consistent international care, improve research comparability, and reconcile historical risk thresholds with modern 3D imaging.

Acute type A aortic dissection is a catastrophic cardiovascular emergency characterized by mortality rates reaching 50% within the first 24 h without surgical intervention.^1^ While elective prophylactic root replacement is the established gold standard for prevention, carrying a low operative mortality of <2.5% at high-volume centres, success depends entirely on accurate risk stratification.^2^ Historically, clinicians have relied on maximal aortic diameter as the primary biomarker for intervention. However, international guidelines lack consensus on measuring the non-circular aortic root: the European Society of Cardiology (ESC) recommends middle cusp-to-cusp measurements (center of a sinus to the opposing sinus), while the European Association for Cardio-Thoracic Surgery (EACTS) suggests the widest cusp-to-cusp technique (nadir of one sinus to the furthest point on the opposing wall) as depicted in *Figure 1*.^3,4^

**Figure 1:**
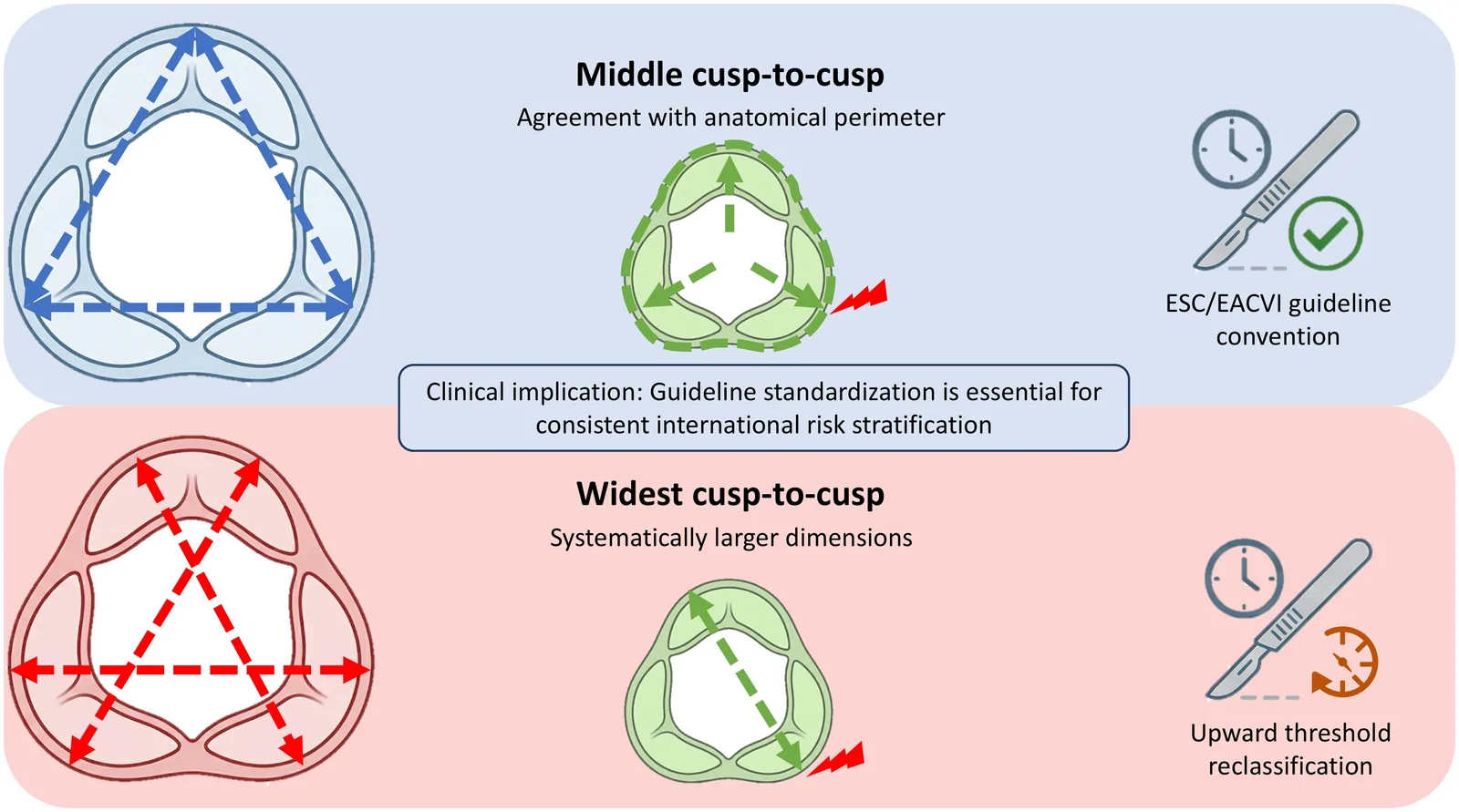
Difference in cusp-to-cusp measurement techniques and potential implications. This image demonstrates the two different measuring techniques and summarizes the impact of these techniques on aortic root wall stress and potential surgical timing.

This investigation was designed as a retrospective, observational study of clinical imaging datasets obtained at Radboud University Hospital. The study protocol was reviewed and approved by the institutional ethical committee. The study cohort consisted of 50 consecutive patients who underwent clinically indicated cardiovascular magnetic resonance (CMR) imaging between August 2024 and January 2025. The population represented a relative young cohort (mean age 42 years) typical of aortic surveillance programmes. To isolate the geometric variables of interest and ensure the applicability of theoretical models, strict exclusion criteria were applied. Patients with bicuspid aortic valves were excluded due to the elliptical asymmetry often associated with this phenotype, which would confound the comparison of standardized tricuspid root measurements. Similarly, patients with prior aortic surgery or existing aortic root dissection were excluded to avoid the distorting effects of surgical remodelling or false lumens on native dimensions.

All imaging measurements were derived from high-resolution, three-dimensional datasets acquired using a non-contrast 3D TRUFI (True Fast Imaging with Steady-State Precession) sequence. Measurements were performed using commercially available post-processing software (Medis). The middle cusp-to-cusp measurements were already available. The widest cusp-to-cusp measurements and the tracing of the perimeter were performed by an operator with 2 years of CMR experience.

To evaluate the relationship between the linear measurements and the perimeter, we developed a theoretical geometric model. We assumed that the cross-section of the aortic root at the level of the sinuses could be approximated by a circle, with the three commissures forming an inscribed equilateral triangle. In this scenario, defining the cusp-to-cusp distance (*d*) as the triangle's side and R as the circumscribed radius yields the relationship R=d/√3. Since the diameter of a circle (*D*) is 2R, it follows that D=2d/√3 resulting in a perimeter $P=\;\pi \;\times \;D=\frac{2\pi d}{\sqrt{3}}\approx 3.628\;\times \;d$. This formula was used to test which of the measured diameters (Widest or Middle) demonstrated greater internal consistency with the traced perimeter.

The primary objective was to evaluate the agreement between the calculated aortic root perimeters (derived from our theoretical model using middle and widest cusp-to-cusp distances) and the traced perimeter. This agreement was quantified using the Lin’s Concordance Correlation Coefficient (CCC) to assess both precision and accuracy. Additionally, the Mean Absolute Error (MAE) and Root Mean Square Error (RMSE) were calculated for each model to determine the average magnitude of the errors. A higher CCC combined with lower MAE and RMSE values indicated a better approximation of the traced perimeter.

Clinical threshold reclassification was evaluated across established guideline cutoffs (40, 45, 50, and 55 mm). Patients were categorized below or at/above each threshold using ESC (middle cusp-to-cusp) vs. EACTS (widest cusp-to-cusp) conventions. Upward reclassification was defined as crossing a threshold when switching from middle to widest measurements. Paired categorical discordance was evaluated using McNemar’s test. Numerical assessment of agreement and bias was performed using Bland-Altman analysis and plots.

Statistical analysis was performed using R version 4.4.2 (R Foundation for Statistical Computing, Vienna, Austria). Continuous variables were assessed for normality to determine the appropriate comparative tests. To compare the differences between specific measurements, paired t-tests were used for normally distributed data, while the Wilcoxon signed-rank test was employed for non-normally distributed data. The packages ‘ggplot2’, ‘tidyr’, ‘patchwork’ and ‘dplyr’ were used for data manipulation and graphical plots. The ‘DescTools’ package was utilized for the Concordance Correlation Coefficient and ‘Metrics’ for MAE and RMSE calculations. The ‘boot’ package was used for calculating confidence intervals and ‘ggsankey’ for the Sankey diagram. A *P*-value of <0.05 was considered statistically significant for all analyses.

Across the cohort, the absolute mean measured diameter was 32.5 ± 6.6 mm using the middle cusp-to-cusp technique and 36.1 ± 7.1 mm using the widest technique, while the traced anatomical perimeter averaged 114.2 ± 23.0 mm. The widest cusp-to-cusp measurement yielded systematically higher values than the middle measurement, with a significant mean difference of 3.60 mm (95% CI: 3.35–3.85, *P* < 0.001). The middle measurement demonstrated superior statistical agreement with the traced aortic root perimeter, achieving a Lin’s Concordance Correlation Coefficient (CCC) of 0.983 (95% CI: 0.97–0.99). Furthermore, the middle measurement produced the lowest error rates, with a Mean Absolute Error (MAE) of 3.96 mm (95% CI: 3.22–4.73) and a Root Mean Square Error (RMSE) of 4.80 mm (95% CI: 3.98–5.58). Conversely, widest cusp-to-cusp measurements showed substantially lower agreement (CCC 0.80, 95% CI: 0.72–0.85) and significantly higher error rates (MAE 16.88 mm, 95% CI: 15.97–17.85). Numerical Bland-Altman limits of agreement (LoA) confirmed that the widest technique consistently overestimates the perimeter (16.88 mm (95% LoA: 10.27–23.50) vs. 3.82 mm (95% LoA: −1.93–9.85); *Figure 2*). Applying the widest cusp-to-cusp technique resulted in upward reclassification across the 40 and 45 mm thresholds in 10% (5/50) and 16% (8/50) of patients, respectively (*Figure 3*).

**Figure 2:**
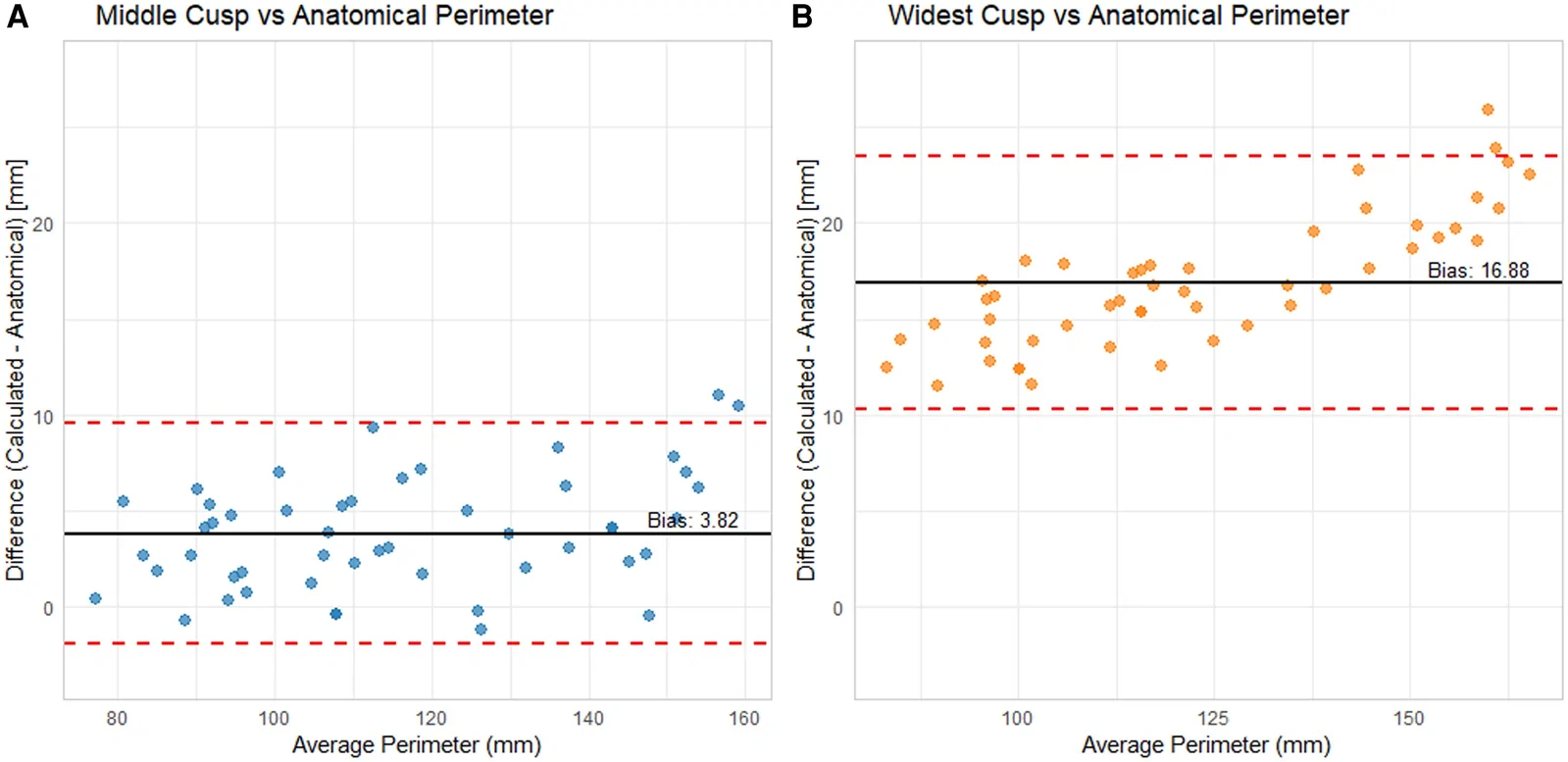
Geometric agreement and clinical threshold reclassification of aortic root measurement techniques. Bland–Altman plots illustrate agreement between the traced anatomical perimeter and calculated perimeters derived from the middle cusp-to-cusp technique (Panel *A*) and the widest cusp-to-cusp technique (Panel *B*), with solid horizontal lines indicating mean bias and dashed lines representing 95% limits of agreement.

**Figure 3:**
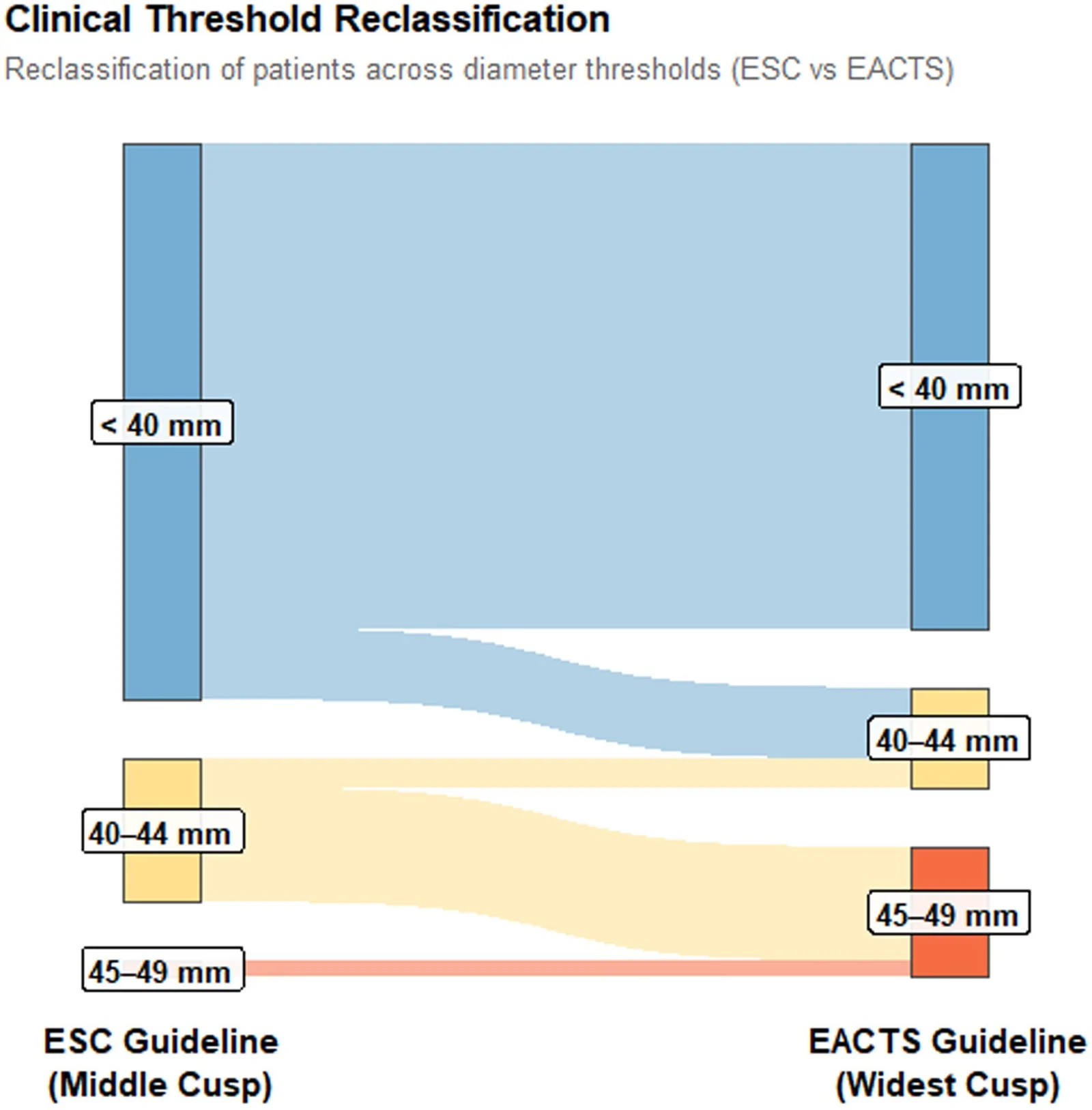
Patient reclassification across aortic root diameter thresholds. Sankey diagram illustrating patient flow across established clinical cutoffs (<40, 40–44, 45–49, and ≥ 50 mm) when comparing the ESC recommendation (middle cusp-to-cusp) with the EACTS recommendation (widest cusp-to-cusp). Connecting flows depict patients remaining in the same tier vs. those undergoing upward reclassification.

The clinical reliance on diameter is rooted in the Law of Laplace, which posits that circumferential wall stress is proportional to the vessel radius.^5^ However, the non-axisymmetric architecture of the aortic root, characterized by three bulging sinuses, violates the assumption of uniform circularity.^6^ Biomechanically, wall stress reaches its zenith where the wall is the thinnest and the local radius of curvature is maximal, which for the sinus is the middle of the cusp.^7^ Anatomically, the wall is flattest (maximal radius of curvature) at the centre of the sinus. We hypothesize that the middle cusp-to-cusp technique targets this region of maximal local wall stress. Furthermore, the middle measurement approximates the perimeter more accurately which also correlates with outcome.^8^

Several limitations of this study should be acknowledged. First, the theoretical geometric model assumes symmetric, equilateral root architecture, which may oversimplify complex geometries in asymmetric sinuses or bicuspid aortic valve phenotypes. Second, our cohort is relatively small and derived from a single tertiary centre without longitudinal follow-up for clinical endpoints. Finally, this study did not incorporate computational fluid dynamics, finite element analysis, or patient-specific tissue testing. Consequently, biomechanical considerations regarding local wall stress remain theoretical hypotheses supported by anatomical curvature principles and literature from abdominal and intracranial aneurysms rather than direct, sinus-specific experimental testing.

Our data demonstrate a directly measured, clinically meaningful divergence between ESC and EACTS measurement conventions, causing substantial threshold discordance. While the closer perimeter agreement of the middle technique serves as a secondary, hypothesis-generating observation, it’s clinical superiority remains to be investigated in future studies. Ultimately, regardless of which convention proves superior in future prospective studies, reconciling international guidelines through a single standardized measurement convention is essential for uniform patient surveillance and risk stratification. Lacking prospective outcome trials, a choice of measurement technique must be made using current evidence until superior predictors of aortic rupture emerge.

## Data Availability

Data are available upon reasonable request.

## Lead author biography

I am a dedicated physician and researcher specializing in Cardiology, with a particular focus on cardiac imaging and adult congenital heart disease (ACHD). Currently, I'm completing a specialized Fellowship in Cardiac Imaging, focusing on MRI, and ACHD at Radboud University Medical Center (Radboud UMC) in Nijmegen, under the guidance of Prof. Dr. R. Nijveldt.

My clinical trajectory includes a Cardiology Residency, with training at both Catharina Hospital in Eindhoven and Radboud UMC, where I developed expertise in state-of-the-art cardiac MRI. This clinical experience is supported by an academic background, highlighted by the completion of my Ph.D. in Cardiology from 2017 to 2021 at the Eindhoven University of Technology (TU/e), Department of Biomedical Engineering. The doctoral research was conducted under the supervision of Prof. Dr. N.H.J. Pijls, Dr. P.A.L. Tonino, and Asc. Prof. N.P. Johnson, focused on invasive and non-invasive (patho-)physiologic responses during exercise in patients with aortic valve stenosis.

Prior to specialized training, I earned both a Master of Medicine (2012–2015) and a Bachelor of Medicine (2009–2012) from Maastricht University’s Faculty of Health, Medicine and Life Sciences, following initial studies in Knowledge Engineering. I hope this blend of physiology, advanced imaging, and congenital heart disease expertise helps me to improve the field of Cardiology both clinically and academically.
